# Characterisation of microbiota in saliva, bronchoalveolar lavage fluid, non-malignant, peritumoural and tumour tissue in non-small cell lung cancer patients: a cross-sectional clinical trial

**DOI:** 10.1186/s12931-020-01392-2

**Published:** 2020-05-25

**Authors:** Rea Bingula, Edith Filaire, Ioana Molnar, Eve Delmas, Jean-Yves Berthon, Marie-Paule Vasson, Annick Bernalier-Donadille, Marc Filaire

**Affiliations:** 1 Université Clermont Auvergne, INRAE, UNH, F-63000 Clermont–Ferrand, France; 2 Greentech SA, Biopole Clermont-Limagne, 63360 Saint-Beauzire, France; 3 Centre Jean Perrin, INSERM, U1240 Imagerie Moléculaire et Stratégies Théranostiques, Université Clermont Auvergne, F-63011 Clermont-Ferrand, France; 4 Délégation Recherche Clinique & Innovation, Centre Jean Perrin, Centre de Lutte contre le Cancer, F-63011 Clermont-Ferrand, France; 5 Centre d’Investigation Clinique, UMR501, F-63001 Clermont-Ferrand, France; 6 Université Clermont Auvergne, INRAE, MEDIS, 63122 Saint-Genes-Champanelle, France; 7 Centre Jean Perrin, CHU Gabriel-Montpied, Clinical Nutrition Unit, F-63000 Clermont-Ferrand, France; 8 Thoracic Surgery Department, Centre Jean Perrin, 63011 Clermont-Ferrand, France

**Keywords:** Lung cancer, Lung microbiota, Non-small cell lung cancer, Bronchoalveolar lavage, Peritumoural lung tissue, Non-malignant lung tissue, Lower lobe tumour, Lobe location, Saliva

## Abstract

**Background:**

While well-characterised on its molecular base, non-small cell lung cancer (NSCLC) and its interaction with local microbiota remains scarcely explored. Moreover, current studies vary in source of lung microbiota, from bronchoalveolar lavage fluid (BAL) to tissue, introducing potentially differing results. Therefore, the objective of this study was to provide detailed characterisation of the oral and multi-source lung microbiota of direct interest in lung cancer research. Since lung tumours in lower lobes (LL) have been associated with decreased survival, characteristics of the microbiota in upper (UL) and lower tumour lobes have also been examined.

**Methods:**

Using 16S rRNA gene sequencing technology, we analysed microbiota in saliva, BAL (obtained directly on excised lobe), non-malignant, peritumoural and tumour tissue from 18 NSCLC patients eligible for surgical treatment. Detailed taxonomy, diversity and core members were provided for each microbiota, with analysis of differential abundance on all taxonomical levels (zero-inflated binomial general linear model with Benjamini-Hochberg correction), between samples and lobe locations.

**Results:**

Diversity and differential abundance analysis showed clear separation of oral and lung microbiota, but more importantly, of BAL and lung tissue microbiota. Phylum *Proteobacteria* dominated tissue samples, while *Firmicutes* was more abundant in BAL and saliva (with class *Clostridia* and *Bacilli*, respectively). However, all samples showed increased abundance of phylum *Firmicutes* in LL, with decrease in *Proteobacteria*. Also, clades *Actinobacteria* and *Flavobacteriia* showed inverse abundance between BAL and extratumoural tissues depending on the lobe location. While tumour microbiota seemed the least affected by location, peritumoural tissue showed the highest susceptibility with markedly increased similarity to BAL microbiota in UL. Differences between the three lung tissues were however very limited.

**Conclusions:**

Our results confirm that BAL harbours unique lung microbiota and emphasise the importance of the sample choice for lung microbiota analysis. Further, limited differences between the tissues indicate that different local tumour-related factors, such as tumour type, stage or associated immunity, might be the ones responsible for microbiota-shaping effect. Finally, the “shift” towards *Firmicutes* in LL might be a sign of increased pathogenicity, as suggested in similar malignancies, and connected to worse prognosis of the LL tumours.

**Trial registration:**

ClinicalTrials.gov ID: NCT03068663. Registered February 27, 2017.

## Background

Despite the advancements in its detection and treatment, lung cancer (LC) is still the leading cause of death by cancer worldwide [1]. Non-small cell lung cancer (NSCLC) is diagnosed in 85–90% of LC cases and presents the most frequent type of lung cancer. Unlike small cell lung cancer, NSCLC is operable in 20–25% of cases. This concerns mostly early stage tumours (stage I and II), sometimes locally advanced disease (stage III) and rarely oligometastatic disease (stage IV). Other treatments, such as chemotherapy, radiotherapy and until recently immunotherapy, are often associated with surgery as multimodality treatment. Even though surgery is recognised as the most effective initial treatment of NSCLC, the 5-year survival rates remain however low (~ 90% for stage IA1, and ~ 12% for stage IIIC) [2, 3]. Therefore, the tumour staging is used as the important prognostic tool based on tumour size, lymph node invasion and metastatic status [2]. Curiously, tumour lobe location has also been associated to tumours’ aggressiveness, with tumours in lower lobes (LL) showing worse term and 5-year survival after resection than the ones in upper lobes (UL), still without a clear explanation [4–6].

Increasing interest in the interaction between host and its microbiota revealed its potential implication in health and disease, but also in tumour immunology and physiology [7–11]. Certain bacteria, such as genus *Bifidobacterium* [8] or species *Enterococcus hirae* [10], have been found to improve the efficiency of chemotherapy or immune-checkpoint inhibitors if administered orally in animal models. This phenomenon has been explained by their translocation from the gut to mesenteric lymph nodes, the priming of the upstream regulatory immune cells, such as dendritic cells, and causing increased reactivity against tumour epitopes [12, 13]. Moreover, administration of *Bifidobacterium* cocktail alone has been proved equally effective as the anti-PD-1 (Programmed cell Death protein 1) antibody in abolishing tumour growth in the animal melanoma model [8]. Finally, faecal transplantation from the patients responding (enriched in *Bifidobacterium, Akkermansia, Faecalibacterium*) or not (enriched in *Bacteroidales*) to the anti-cancer treatment has induced the same kind of response in receiving tumour-bearing animals [8, 14]. These findings have been one of the most elucidating in terms of interactions between the host’s immune system, gut microbiota and cancer.

Unlike local and systemic effects of the gut microbiota, the lung microbiota and its effects remain scarcely explored, being only recently accepted as one of the resident microbiota (and not only present during infection) [15, 16]. Since then, an emerging number of studies turned to its exploration, notably in the context of cystic fibrosis, asthma and chronic obstructive pulmonary disease (COPD), interstitial lung disease, and lung transplantation [17–22]. Despite its impact on global cancer-related death, lung cancer studies were surprisingly few and started to emerge only a few years ago. However, they confirmed that lung microbiota interacts with local immunity and modifies tumour properties. The microbial dysbiosis in antibiotic-treated or germ-free animals influenced growth of injected lung tumour cells [23, 24] while usage of penicillin, cephalosporins, or macrolides showed increasing risk of lung cancer in human subjects [25]. In lung cancer patients, lung microbiota from bronchoalveolar lavage fluid (BAL) enriched with supraglottic taxa was associated with pro-inflammatory profile and stimulation of Th17 cells with protumourigenic effect [26–28], and also exhibited different abundance and metabolic profiles compared to those of healthy subjects [29, 30]. Interestingly, salivary microbiota was also found to show cancer specific profile, with genera *Veillonella* and *Capnocytophaga* more abundant in saliva of lung cancer patients [31]. At the present, only two studies analysed lung tissue microbiota in lung cancer. One found increased alpha diversity in non-malignant tissue compared to tumours as well as in adenocarcinoma compared to squamous cell carcinoma [32], while the other showed association between increased diversity of the non-malignant tissue (but not tumour) and decreased recurrence-free and disease-free survival [33]. Among studies on lung microbiota, those on BAL are the most numerous, since it remains the sample with acceptable ratio of contamination risk by upper airways, precision in lung microbiota sampling and invasiveness. However, this has been a potential source of contradictory information since varying characteristics of BAL and tissue microbiota, as a result of samples’ different nature, have been previously suggested [21]. Therefore, there has been an increasing necessity to characterise the ground differences between different lung microbiota in NSCLC patients to enable better comprehension of the obtained results depending on the initial lung sample.

As its primary objective, this cross-sectional pilot study analysed lung microbiota from four different samples in 18 NSCLC patients eligible for surgery without neoadjuvant therapy. Lung microbiota was analysed in BAL, non-malignant tissue, peritumoural tissue and tumour, as each sample should have different architectural and physiological characteristics. Unlike what was previously seen, in this study BAL was obtained directly from the excised lobe without passing through the upper airways to decrease the contamination risk. In addition to lung microbiota, salivary microbiota was characterised for each patient and used as an extra-pulmonary sample to put in perspective the relation with and between lung samples. As a second objective, we investigated whether tumour location in the UL or LL yields significant changes in these microbiota.

## Methods and patients

### Patient recruitment and study design

All patients were enrolled in a prospective study, approved by the CPP Sud Est VI Ethics Committee and registered at ClinicalTrials.gov (NCT03068663) [34]. Written informed consent was obtained from all patients before enrolment in the study and any study procedure.

Patients diagnosed with primary NSCLC eligible for surgical treatment with or without neoadjuvant therapy and presented before the Thoracic Oncologic Committee of the Centre Jean Perrin (Clermont-Ferrand, France) were preconsidered for inclusion to the study. Inclusion criteria were: age between 18 and 80 years, body mass index (BMI) < 29.9, no antibiotics, corticoids, immunosuppressive drugs or having undergone pulmonary infections for at least the past 2 months, as well as no previous airway surgery or cancer treatment. Only patients included in the group of patients eligible for surgery without chemotherapy were taken into account in this manuscript.

At inclusion, patients received the tube for saliva collection and were asked to bring it with them the day of their hospital admission for surgery. Sampling of the lung was performed during the surgery immediately after excision of the tumour lobe, representing no additional inconvenience for patients apart from this standard medical procedure. Detailed inclusion/exclusion criteria, the study flowchart as well as detailed design and power calculation were previously published [34].

### Sampling

#### Saliva

Saliva was collected as previously described [34]. Upon reception by the study personnel, the sample was stored at − 80 °C until DNA extraction.

#### Lung tissue and BAL

Sampling of lung tissue and BAL during surgery was performed immediately after partial or complete pneumonectomy. The removed lung or lung lobe was placed in a sterile vessel and the tumour position was determined by palpation. First, a piece of non-malignant lung distal to the tumour (opposite side of the lobe) with an average size of 1 cm^3^ was clamped. The clamp was left in place until the end of the following procedure. Using a sterile syringe, the excised lung was inflated through the main bronchus. Bronchoalveolar lavage was performed by instilling 2 × 40 mL of sterile physiological saline into the bronchus. After each instillation, the maximum amount of liquid inside the bronchus was retrieved (8–10 mL in total) into 50 mL tube (designated as “BAL”). Then, the clamped wedge of non-malignant tissue was cut off and designated as LUNG.DP (“distal piece”). Further, a pie-slice of the tumour (cross-section) was excised with its peritumoural tissue, after which the two were separated based on macroscopic histological difference. Tumour tissue sample was designated as “LUNG.T” and peritumoural tissue sample as “LUNG.PT”. The tissues were snap-frozen in liquid nitrogen and then placed at − 80 °C for long-term storage until DNA extraction. BAL was stored directly at − 80 °C.

### DNA extraction and negative controls

DNA was extracted as previously described [34]. Saliva and BAL (cellular BAL) volume used for DNA extraction were 1 and 5 mL, respectively. Initial tissue weights ranged from 377 ± 236 mg for LUNG.PT, to 1.441 ± 1.016 g and 1.346 ± 0.899 g for LUNG.DP and LUNG.T. Even though the initial weight between tissue samples was significantly different (*p* = 0.001), there was no difference in final concentration of DNA/g of sample (*p* = 0.895). DNA extraction from lung tissue samples (three samples per patient) was randomised (each extraction group never contained only one sample type or all samples from the same patient) to randomise the manipulation effect.

Since the lung samples are considered as low biomass samples, background controls were made throughout the sampling and extraction procedure. Negative sampling control was collected for each BAL and consisted of physiological serum collected with syringe used for instillation from the same liquid recipient. During the DNA extraction, milliQ water was used as a negative background control and underwent the same procedure as the real samples. All controls were sequenced and analysed. Reagents used in DNA extraction and sample pre-treatments were either autoclaved, filtered through 20 μm filters or purchased sterile. All tools and pipettes were thoroughly washed and disinfected before and after each extraction cycle or between different extraction steps.

### 16 ribosomal RNA (16S rRNA) gene sequencing

DNAVision (Belgium), using Illumina MiSeq technology, performed 16S ribosomal rRNA gene sequencing. Following the PCR amplification of the targeted region V3-V4, libraries were indexed using the NEXTERA XT Index kit V2. The sequencing was carried out in paired-end sequencing (2 × 250 bp) by targeting an average of 10,000 reads per sample. Next, sample sequences were clustered into OTUs based on 97% sequence similarity. This was performed with software QIIME (Quantitative Insights Into Microbial Ecology). Further microbiota analyses were done on generated “raw” OTU (outer taxonomic unit) table, taxonomy and Newick formatted phylogenetic tree provided by DNAVision.

### Sequence processing and microbiota analysis

Microbiota analysis and visualisation were done with RStudio 3.5.2 [35]. (packages “phyloseq” [36], “vegan” [37],“microbiome” [38], “ggplot2” [39], “DESeq2” [40],“metacoder” [41]).

Raw OTU table was filtered to keep only kingdom *Bacteria* for further analysis. The total of 26 negative controls (sampling and background) had in average 21 detected OTU with average of 4 reads/OTU and did not belong to more abundant OTUs in samples. These OTU counts were subtracted from corresponding samples, i.e. negative sampling control (physiological serum for washing) from corresponding BAL sample, and negative extraction controls from the samples in the extraction group. Samples were processed in two batches with controlled randomisation of all sample types and patients, including clinical data, so that both batches were equally diverse. Therefore, only OTUs present in all samples of one batch and not present in all samples of the other were excluded from further processing as a consequence of unequal extraction efficiency (and not of contamination). These preprocessings did not alter any of the measures, and have left the data virtually unchanged. Next, OTU present in at least 10% of the lung samples and 20% of saliva samples or having more than 50 overall counts (for each group) were kept for further processing (in our case, this was equivalent of keeping the OTUs with minimal average abundance of 0.001% in either of groups). Only samples with more than 1000 reads were included in analysis. This excluded 6 samples (without preference for certain factor): 2 BAL, 3 peritumoural tissues (LUNG.PT) and 1 tumour (LUNG.T). Average read number of final sample groups was 39,083 ± 9697 (mean ± SD) for saliva, 17,046 ± 14,879 for BAL, 13,352 ± 12,909 for LUNG.DP, 13,039 ± 11,394 for LUNG.PT and 5846 ± 4505 for LUNG.T.

For analysis of alpha and beta diversity, samples were rarefied at 1195 reads with 100 iterations. Observed OTU number, Shannon diversity index and Faith’s phylogenetic diversity were used for alpha diversity characterisation (“phyloseq”). Groups’ beta diversity was calculated based on weighted (importance of abundance and quality) (wUF) and unweighted (importance of absence or presence of OTUs) (uwUF) UniFrac distances using function adonis (“vegan”) with 999 permutations and presented with non-metric multidimensional scaling (NMDS). Core microbiota clustering was based on Bray-Curtis distance and NMDS method.

Taxonomic trees (“metacoder”) representing relative abundance of taxa within each sample group were based on arithmetic mean of relative abundance calculated from unrarefied OTU table for each group. Input for trees representing differential abundance was calculated by DESeq function using zero-inflated method of negative binomial general linear model (significant coefficient difference calculated by Wald’s test) and Benjamini-Hochberg (BH) correction for multiple comparison with 0.05 threshold (“DESeq2”). The model was used on unrarefied taxa counts.

Difference in alpha diversity and paired UF distances was calculated by Kruskal-Wallis or Man-Whitney U test with BH correction for multiple comparison with 0.05 threshold of significance.

### Managing missing data – paired/unpaired tests

A total of six lung samples was excluded from the study due to insufficient number of reads (< 1000). Excluded samples did not originate from only one specific tissue, only one patient or exclusively belonged to one criterion (only one sex, lobe location, or tumour type). Therefore, preservation of strictly paired analysis would exclude an important number of other related samples. For this reason, analysis was unpaired if not specified otherwise.

## Results

### Participant characteristics

The total of 18 patients eligible to surgical treatment without neoadjuvant therapy was included in the study. Microbiota was analysed in 17 saliva and 68 lung samples, where 16 patients provided all 5 different samples (1 saliva plus 4 lung). For the analysis relative to the location of the tumour lobe, patients were grouped into two groups: 1st group with tumour in upper lobes (UL), and 2nd group with tumour in middle and lower lobes (LL) (share same descending bronchus). Patients with LL tumour had significantly lower predicted diffusing capacity of the lung for carbon monoxide (DLCO) than patients with tumours in UL (*p* = 0.034). Other clinical parameters showed no significant difference. Patients’ characteristics (total and per lobe location group) and final sample number used in analysis after exclusion of samples with less than 1000 reads are shown in Table 1.

**Table 1:** Characteristics of patients included in the study

	Total	Upper lobe T	Lower lobe T	*p*
No. of patients	18	10	8	
Male/female	13/5	8/2	5/3	
Age (years)	68 ± 8	65 ± 9	72 ± 6	0.061
BMI	25 ± 3	25 ± 4	25 ± 3	0.859
T in upper/middle/lower lobe	10/2/6	10/0/0	0/2/6	
ADC/SCC/carcinoid	11/5/2	6/2/2	5/3/0	
Stage I/II/III	8/2/8	5/2/3	3/0/5	
Tumour size (cm)	3.7 ± 2.3	3.6 ± 2.4	3.9 ± 2.4	1
Smoker/ex-smoker/never-smoker	2/14/2	0/9/1	2/5/1	
Pack-year (smokers, ex-smokers)	31 ± 20	31 ± 21	33 ± 20	0.823
FEV_1_ (% of expected value)	98 ± 11	95 ± 8	101 ± 14	0.408
DLCO (% of expected value)	74 ± 16	81 ± 13	64 ± 16	**0.034**
FEV_1_/FVC (% of expected value)	96 ± 10	95 ± 11	98 ± 10	0.630
Final no. of analysed samples				
Saliva	17	10	7	
BAL	15	8	7	
LUNG.DP	17	10	7	
LUNG.PT	14	9	5	
LUNG.T	16	9	7	

*ADC* adenocarcinoma, *BAL* bronchoalveolar lavage fluid, *BMI* body mass index, *DLCO* diffusing capacity of the lung for carbon monoxide, *FEV*_*1*_ forced expiratory volume per second, *FVC* forced vital capacity, *LUNG.DP* non-malignant tissue, *LUNG.PT* peritumoural tissue, *LUNG. T* tumour, *SCC* squamous cell carcinoma, *T* tumour

### Beta diversity identifies BAL as a unique sample

**Fig. 1:**
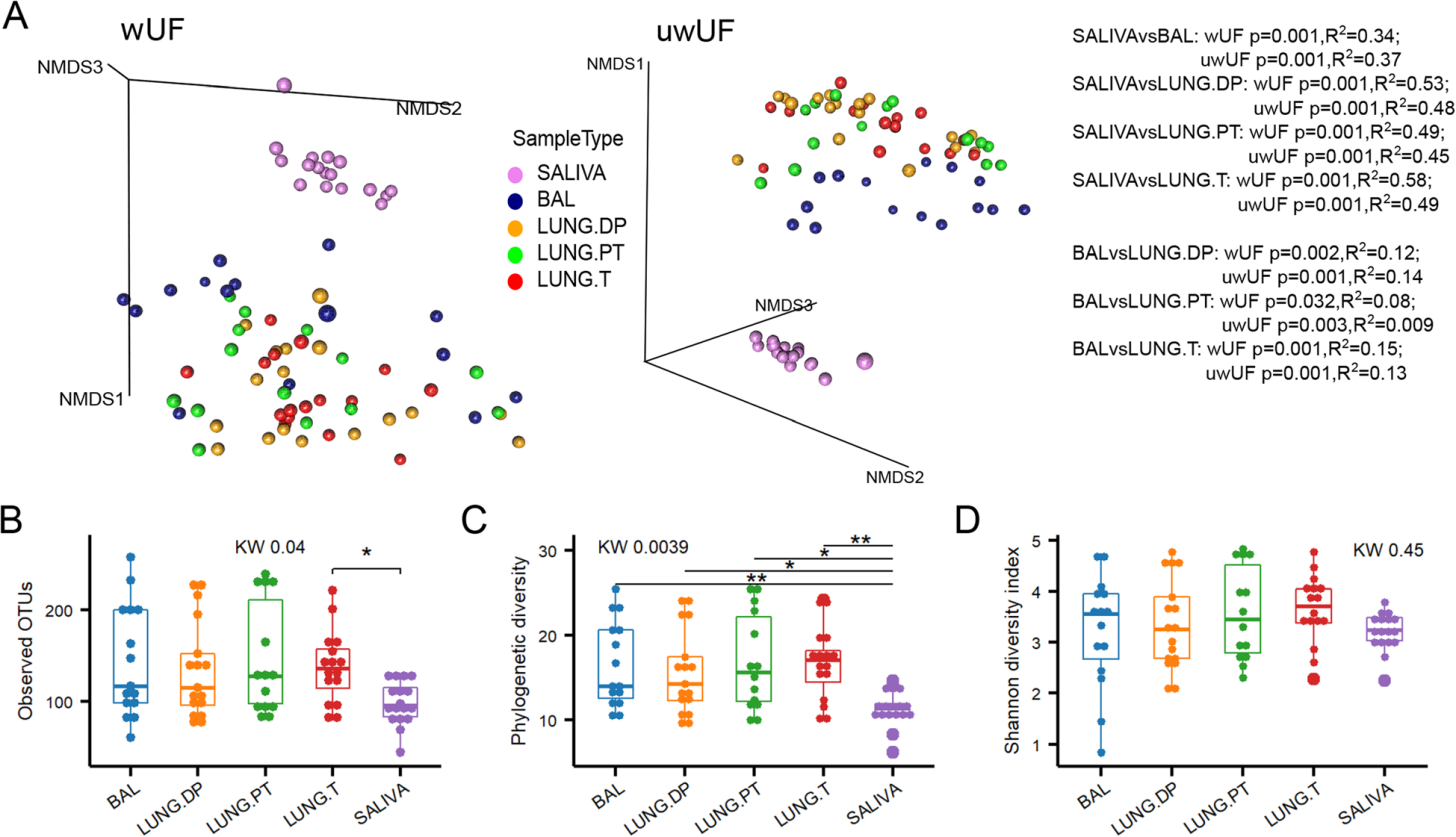
Diversity of the salivary and four lung microbiota. **a** Beta diversity of salivary and lung microbiota represented by non-metric multidimensional scaling (NMDS) based on weighted and unweighted UniFrac distances. Alpha diversity of saliva and four lung samples assessed by **b** number of observed OTUs, **c** Faith’s phylogenetic diversity, and **d** Shannon diversity. Statistical significance of difference in beta diversity was assessed with adonis function (vegan) with 999 permutations. Statistical significance of difference in alpha diversity was assessed with Kruskal-Wallis followed by, where appropriate, Man-Whitney U test with BH correction for multiple comparison. *: *p* ≤ 0.05, **: *p* ≤ 0.01. BAL - bronchoalveolar lavage fluid, KW – Kruskal-Wallis test, LUNG.DP - non-malignant distal piece, LUNG.PT - peritumoural tissue, LUNG. T – tumour, uwUF – unweighted UniFrac, wUF – weighted UniFrac

Microbiota was analysed in saliva, bronchoalveolar lavage fluid (BAL), non-malignant Distal Piece (LUNG.DP), Peritumoural Tissue (LUNG.PT) and Tumour (LUNG.T). As expected, saliva showed a clear separation from the four lung samples (BAL and tissues) with significant difference in beta diversity based on both weighted and unweighted UniFrac distances (Fig. 1a). Moreover, the three lung tissues were all significantly different from BAL (Fig. 1a), but with peritumoural tissue showing the least significant dissimilarity (wUF). There was, however, no significant difference between tissues. Looking at samples’ position in NMDS (Fig. 1a), it was visible that BAL creates a distinct cluster with other three lung tissues when compared to salivary microbiota. However, within this “lung” cluster, BAL samples were concentrated on the front towards saliva cluster.

The four lung samples shared similar average values of observed OTU number (~ 120) as well as phylogenetic (~ 15) and Shannon diversity indexes (~ 3.5) (Fig. 1b-d). Although, compared to saliva, lung microbiota had higher variance, all lung samples showed significantly higher phylogenetic diversity and higher number of observed OTUs (latter significant only for tumour) compared to salivary microbiota. However, there was no difference in Shannon diversity between saliva and lung samples, with averaging index value of ~ 3.5 (Fig. 1d).

### *Proteobacteria* and *Firmicutes* (class *Clostridia*) dominate lung samples

**Fig. 2:**
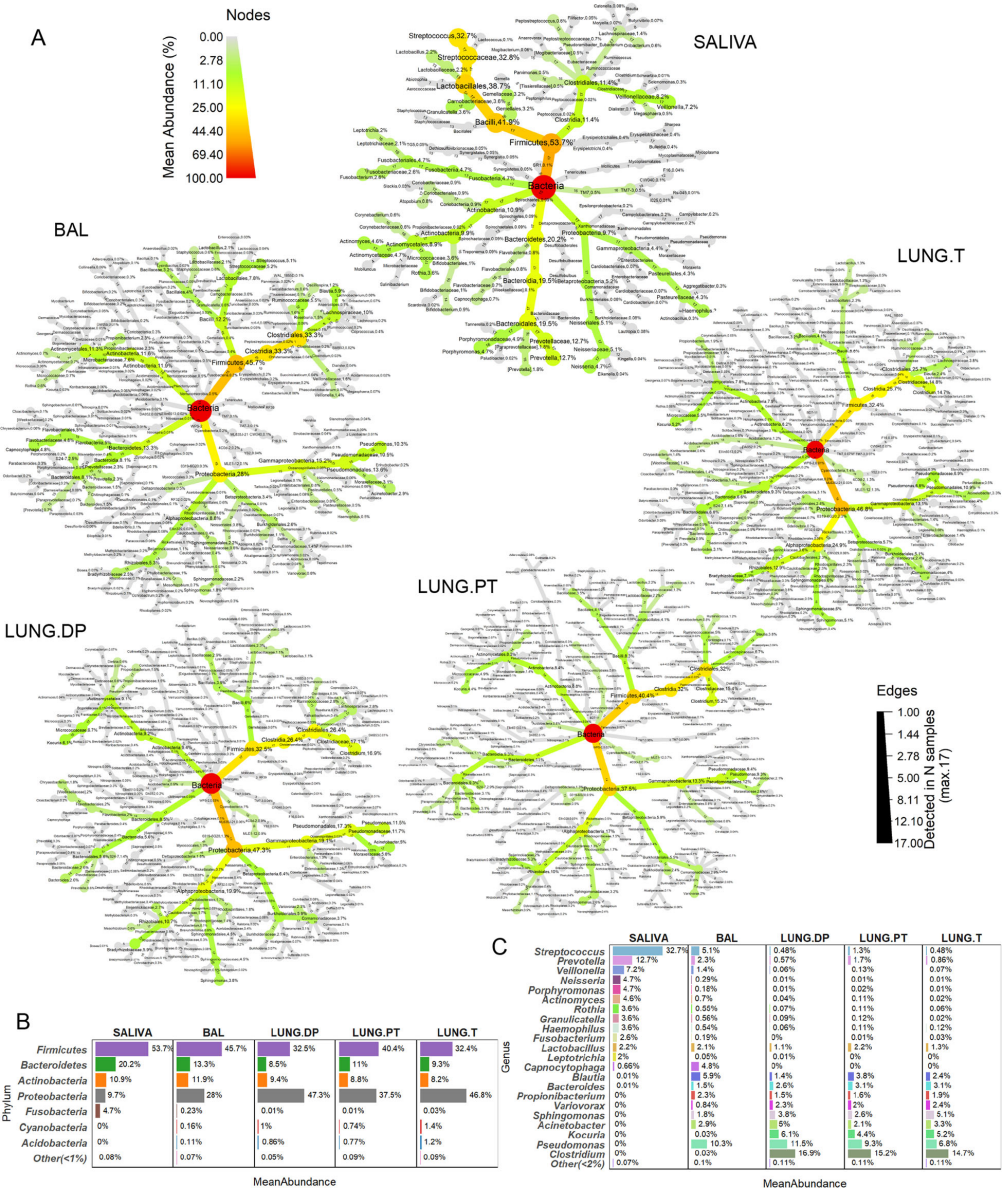
Relative abundance and prevalence of the four lung and salivary microbiota. **a** Each tree represents the taxonomical composition of one sample type. The colour and node size correspond to taxon abundance. All taxa with abundance higher than 0.001% are shown, and percentage is noted for all taxa with abundance higher than 0.01%. Number of samples within which the taxon was detected is noted within branches. Maximal number of samples is 17 for saliva and non-malignant tissue, 16 for tumour, 15 for BAL, and 14 for peritumoural tissue (Table 1). Synthetic presentation of the most abundant taxa was provided on **b** phylum and **c** genus level. BAL - bronchoalveolar lavage fluid, LUNG.DP - non-malignant distal piece, LUNG.PT - peritumoural tissue, LUNG. T - tumour

Thirteen phyla, 29 classes (27 in BAL), 87 families (85 in BAL and tumour), and between 112 and 115 genera were detected in each of the lung samples. In saliva these numbers were lower, with 10 phyla, 17 classes, 26 orders, 49 families and 68 genera. Composition of each sample is shown in Fig. 2a in a form of a taxonomic tree with indicated average relative abundance of taxa next to taxon’s name (> 0.001%) and the number of samples in which they were detected (numbers within branches). The most abundant phyla and genera in each sample are synthetically presented in Fig. 2b and c, respectively. The saliva tree was the least complex from all the trees, heavily dominated by the phylum *Firmicutes* (53.7%) and belonging to genus *Streptococcus* (32.7%) (Fig. 2a, Fig. 2b). Other phyla, as *Bacteroidetes*, *Actinobacteria*, *Proteobacteria* and *Fusobacteria*, were less abundant and are noted in the decreasing order (Fig. 2b). Except *Streptococcus*, additional 11 genera had abundance higher than 1%, including *Prevotella*, *Veillonella*, *Neisseria*, *Porphyromonas*, and *Actinomyces* as the top five.

In the lung samples, two most abundant phyla were *Proteobacteria* and *Firmicutes,* but the dominating one changed relative to the sample*.* So, phylum *Firmicutes* was the most abundant in BAL, while *Proteobacteria* dominated non-malignant tissue and tumour (Fig. 2a, b). Interestingly, in peritumoural tissue two phyla were equally abundant. Phyla *Bacteroidetes* and *Actinobacteria* were found in lower abundance in all lung samples (~ 10% each). Interestingly, while high abundance of phylum *Firmicutes* in saliva was almost entirely due to members of the class *Bacilli*, in lung samples it is due to class *Clostridia*, introducing one of the fundamental differences between these two microbiota. Moreover, *Clostridia* was the most abundant class in all lung samples except the tumour, where it shared the highest abundance with the class *Alphaproteobacteria*. Compared to saliva, the whole phylum *Proteobacteria* was more developed in lung samples, containing additional large class of *Alphaproteobacteria,* but lacking *Epsilonproteobacteria* (detected in saliva).

On the genus level, there was no extensive prevalence by one genus as seen in saliva, but rather a group of representatives with different taxonomic origin (Fig. 2a, c). In the three lung tissues, *Pseudomonas*, *Clostridium*, *Kocuria*, *Acinetobacter* and *Sphingomonas* were the five most abundant genera, but in BAL, those were *Pseudomonas, Blautia, Streptococcus, Capnocytophaga* and *Acinetobacter* (Fig. 2c). Interestingly, two highly abundant genera in tissues, *Clostridium* (~ 15%) and *Kocuria* (~ 5%) were found in very low abundance in BAL. Inverse was seen for *Capnocytophaga*, that seemed to be a BAL-related genus (~ 5%), with a very low presence in saliva and absence in tissue samples. Furthermore, BAL was the only lung sample that had higher abundance of so-called supraglottic taxa, as *Streptococcus*, *Prevotella* and *Veillonella,* compared to other tissue samples. The abundance was also slightly higher in peritumoural tissue, supporting potentially increased similarity between BAL and peritumoural tissue seen in beta diversity (Fig. 1a).

### Whole phyla and classes significantly different between lung samples and saliva, but also between BAL and tissues

**Fig. 3:**
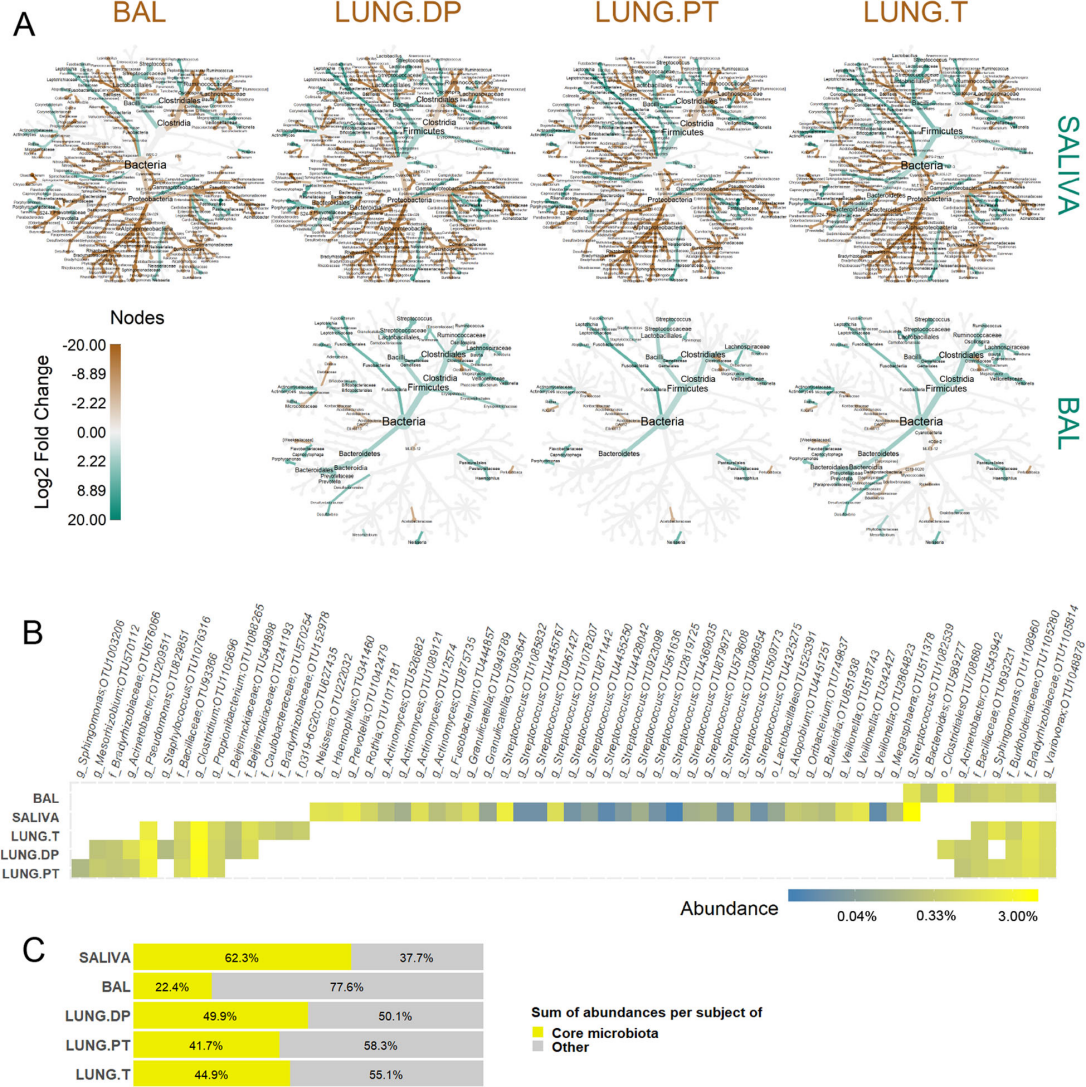
Differential abundance between lung and salivary microbiota and their core composition. **a** Coloured nodes and branches in each tree represent the taxa with significantly different abundance between two compared microbiota. The colour intensity is proportional to log2-fold change in abundance in the favour of the sample with the same colour. Taxa names are shown in the common legend tree below comparisons. Statistical significance was assessed by zero-inflated general linear model using Wald’s test (DESeq2), with *p*-value threshold of α ≤ 0.05 after BH correction. **b** Core microbiota determined as OTUs present in 100% of each sample for one sample type. The colour represents relative abundance on transformed log4 scale. **c** Average value of sum of abundances of the core OTUs and of other OTUs per each subject in each sample types. BAL - bronchoalveolar lavage fluid, LUNG.DP - non-malignant distal piece, LUNG.PT - peritumoural tissue, LUNG. T – tumour, OTU – outer taxonomic unit

Figure 3a shows difference in taxa abundance between samples. Taxa with significant difference are coloured, with a colour scale representing log2 fold change in abundance between compared sample pair.

The first row (Fig. 3a) shows taxa with significantly different abundance between saliva and each of the four lung samples. Compared to saliva, all lung samples had significantly higher abundance in whole classes of *Alphaproteobacteria, Deltaproteobacteria, Cytophagia, Sphingobacteriia, [Saprospirae],* and *Acidimicrobiia*, but also in whole phyla such as *Cyanobacteria, Acidobacteria, Nitrospirae, Verrucomicrobia* and *Planctomycetes* (latter not seen for BAL). Similarly, phyla *Synergistetes, Spirochaetes, Fusobacteria* and *TM7* (not in BAL), classes *Epsilonproteobacteria* and *Erysipelotrichi* were significantly more abundant in saliva than in any of lung samples. On the other hand, multiple descending members of several higher taxa were not strictly more present in only one sample type. This particularly concerned members in the classes from the principal phyla *Proteobacteria, Firmicutes, Bacteroidetes* and *Actinobacteria*. Significantly more abundant in saliva samples were orders *Neisseriales* (*Betaproteobacteria*), *Pasteurellales* (*Gammaproteobacteria*, contains *Haemophilus*), *Lactobacillales* (*Bacilli*, contains *Streptococcus*) and families *Veillonellaceae* (*Clostridia*), *Flavobacteriaceae* (*Flavobacteriia*), *Prevotellacea*e, *Porphyromonadaceae* (both *Bacteroidia*), *Actinomycetaceae* and *Corynebacteriaceae* (both *Actinobacteria*). Conversely, from the same higher taxa, significantly more abundant in lung samples were orders *Burkholderiales* (*Betaproteobacteria*), *Pseudomonadales*, *Legionellales*, *Xanthomonadale*s, *Enterobacteriales* (both *Gammaproteobacteria*), *Bacillales*, *Turicibacteraceae* (both *Bacilli*), and families *Ruminococcaceae*, *Lachnospiraceae* (both *Clostridia*), *Bacteroidaceae* (*Bacteroidia*), *Propionibacteriaceae*, *Dietziaceae* and *Bogorellaceae* (*Actinobacteria*).

Second row in Fig. 3a shows significant difference in abundance between BAL and the three lung tissues. With a few pair-reserved exceptions, pattern was highly similar between comparisons. Also, it was visible that most of the differences indicated significantly increased abundance in BAL compared to tissues. In BAL, significantly higher abundance was seen in phylum *Fusobacteria*, classes *Clostridia* and *Bacilli* (genera *Streptococcus, Veillonella, Roseburia, Oribacterium, Phascolarctobacterium, Parvimonas,* and *Megasphera*), orders *Pasteurellales* (*Haemophilus*) and *Desulfovibrionales*, families *Actynomicetaceae*, *Flavobacteriaceae* (*Capnocytophaga*), and genera *Atopobium*, *Porphyromonas*, *Neisseria*, and *Rothia*. Adversely, the three tissue microbiota had only a few taxa with significantly higher abundance compared to BAL. Those were the whole phylum *Acidobacteria*, families *Acetobacteraceae* and *Clostridiaceae* (genus *Clostridium*), and genus *Perlucidibaca*.

However, there were differences in taxa that could be observed only between certain BAL-tissue pairs. Interestingly, the highest number of individual differences was seen in comparison between BAL and tumour microbiota. Here, only genus *Coprococcus* was significantly more abundant in BAL, while genus *Kocuria*, orders *Bdellovibrionales, Myxococcales, Rickettsiales*, and class [*Saprospirae*] were all significantly more abundant in tumour. Considering non-malignant tissue microbiota, only family *Dietziaceae* was more abundant, while orders *Bifidobacteriales* and *Erysipelotrichales* were significantly more abundant in BAL. Interestingly, tumour and non-malignant tissue had important number of similar differences in comparison to BAL. While BAL had higher abundance of genera *Blautia, Granulicatella, Ruminococcus, Oscillospira, Prevotella,* and *Mezorhizobium*, more abundant in both tumour and non-malignant tissue were phylum *Cyanobacteria* and family *[Weeksellaceae]*. On the contrary, peritumoural tissue did not share any of these differences with BAL as did the other two tissues. In individual differences, *Kocuria* was the only significantly more abundant genus in peritumoural tissue, and inversely, only *Staphylococcus* was more abundant in BAL.

There were, however, no significant differences between three tissues.

### Core OTUs in lung samples mostly members of phylum *Proteobacteria*

Core microbiota was determined as OTUs detected in 100% of samples in each group (Fig. 3b). The highest number of core OTUs was observed in saliva, with the total of 36. Two-fold less was seen in non-malignant tissue (16), peritumoural tissue (14) and tumour (14), and four-fold less in BAL (9). 75% of core OTUs in saliva belonged to phylum *Firmicutes*, with as high as 17/20 OTUs from genus *Streptococcus*, while additional 15% was from the phylum *Actinobacteria* (especially genus *Actinomyces*). In lung samples, 70% of the core OTUs belonged to the phylum *Proteobacteria* (1/3 from class *Alphaproteobacteria*) and other 30% to *Firmicutes*. Core OTUs were mostly shared between different lung sample types, especially between tissues. OTUs corresponding to genus *Variovorax* and unclassified members of families *Bradyrhyzobiaceae, Burkholderiaceae*, and *Bacillaceae* were detected in all four lung microbiota, while OTUs for genera *Pseudomonas*, *Clostridium* and *Propionibacterium* were only common in all lung tissue microbiota. Even though 30% of lung core OTUs belonged to *Firmicutes*, only one OTU corresponded to genus *Streptococcus* and was a part of BAL core microbiota. This was also the only core OTU shared between saliva and lung samples, i.e. only BAL, which were otherwise clearly distinct. Within core microbiota (Fig. 3b), certain OTUs were uniquely associated with species, such as OTUs for *Rothia mucilaginosa, Propionibacterium acnes, Staphylococcus epidermidis, Prevotella melaninogenica, Variovorax paradoxus, Veillonella parvula* (OTU 518743) and *Veillonella dispar* (other two OTUs). On average (Fig. 3c), core microbiota represented 62% of relative abundance in saliva, against only 22% seen in BAL. In the tissues, the core microbiota represented around half of the total abundance, ranging from 42 to 50% of abundance.

### Microbiota in lower lobes with higher abundance of *Firmicutes* and the diversity of peritumoural tissue as the most influenced by location

**Fig. 4:**
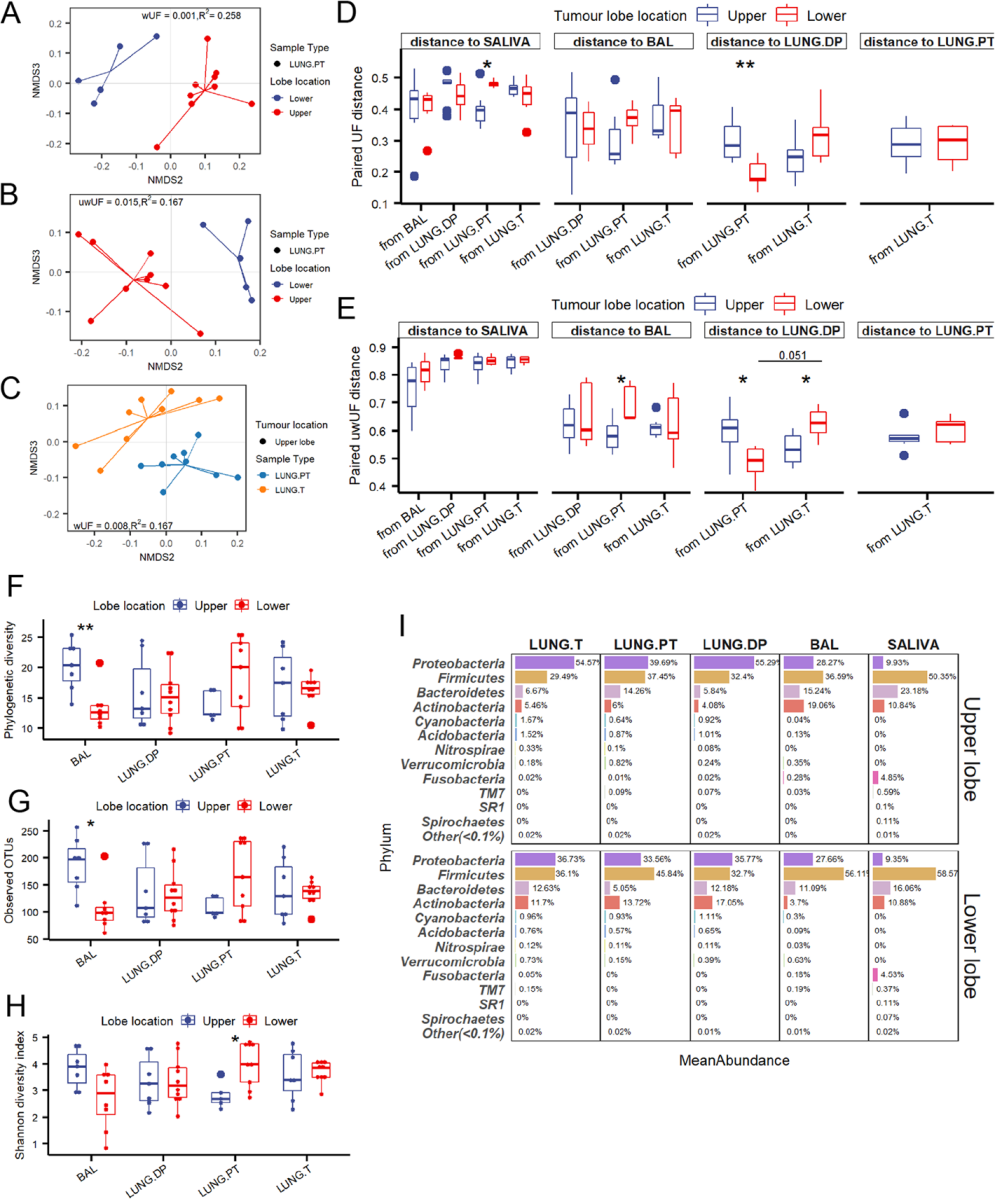
Diversity and predominant taxa in lung samples from upper and lower tumour lobes. Beta diversity found significantly different between peritumoural tissue from upper and lower lobes based on both **a** weighted (wUF) and **b** unweighted (uwUF) UniFrac distances. **c** Significantly different beta diversity based on wUF between peritumoural tissue and tumour in the upper lobe. **d** Weighted and **e** unweighted UF distances between samples coming from the same patient (i.e. paired distances) compared between upper and lower tumour lobes. The facet name represents the referent sample (e.g. “distance to BAL”) to which were calculated the distances noted on x-axis (e.g. “from LUNG.T”). Smaller distance indicates increased similarity. Alpha diversity for four lung samples between upper and lower tumour lobe assessed by **f** Faith’s phylogenetic diversity, **g** number of observed OTUs, and **h** Shannon diversity. **i** Most abundant phyla in each of the microbiota samples if the tumour is found in upper or lower lobes. Significance of difference in beta diversity was assessed with adonis function (vegan, 999 permutations). Statistical significance in alpha diversity and paired distances was assessed with Kruskal-Wallis followed by, where appropriate, Man-Whitney U test with BH correction for multiple comparison. *: *p* ≤ 0.05, **: *p* ≤ 0.01. BAL - bronchoalveolar lavage fluid, LUNG.DP - non-malignant distal piece, LUNG.PT - peritumoural tissue, LUNG. T - tumour

Next, we examined whether there is a significant difference between lung microbiota associated to the tumour lobe relative to its location. Interestingly, only peritumoural tissue microbiota showed significantly different beta diversity between two locations in both wUF and uwUF (Fig. 4a, b). Moreover, difference in beta diversity between UL peritumoural tissue and BAL was not significant, unlike the one in LL (in UL: wUF *p* = 0.17, uwUF *p* = 0.073, vs. in LL: wUF *p* = 0.004, uwUF *p* = 0.002). To confirm this observation, we selected exclusively distances between two samples originating from the same patient (Fig. 4d, e), i.e. paired distances. Indeed, in UL peritumoural microbiota was significantly more similar to both saliva and BAL, manifested as shorter distances compared to ones in LL (Fig. 4d, e: “distance to SALIVA”, “distance to BAL”). We next looked at the paired distances between the three tissue samples (Fig. 4d, e: “distance to LUNG.DP”). The paired distance between non-malignant tissue and the other two tissues, respectively, was inverse depending on the lobe location. In UL, there was an increased similarity between non-malignant tissue and tumour, and in LL, between non-malignant and peritumoural tissue. However, paired distance between peritumoural tissue and tumour remained unchanged (Fig. 4d, e: “distance to LUNG.PT”), suggesting a potentially balanced change or exchange of the microbiota maintaining the distance (and the difference) on the same level. In beta diversity however, these two samples were significantly different only in UL (Fig. 4c), while in LL difference was not significant (*p* = 0.077, *R*^*2*^ = 0.173). Altogether, this indicated that in upper lobes peritumoural tissue microbiota was significantly more similar to BAL microbiota, while in lower lobes peritumoural tissue was more similar to tumour microbiota.

Both BAL and peritumoural tissue varied in alpha diversity depending on lobe location, unlike non-malignant tissue and tumour. BAL in LL had significantly lower phylogenetic diversity and number of observed OTUs (Fig. 4f, g) compared to UL BAL, and a tendency seen for Shannon diversity (Fig. 4h). Inversely, LL peritumoural tissue had significantly increased Shannon diversity (Fig. 4h), with tendency in phylogenetic diversity and number of OTUs.

In LL, there was a marked decrease in abundance of *Proteobacteria* and increase in phylum *Firmicutes* in each of the lung samples (Fig. 4i). Therefore, LL tumour and non-malignant tissue had equal abundances in *Proteobacteria* and *Firmicutes* (~ 35%), while BAL and peritumoural tissue were both dominated by *Firmicutes* (56 and 45%, respectively).

### *Actinobacteria* and *Flavobacteriia* show inverse abundance between BAL and extratumoural tissues depending on the lobe location, while tumour microbiota remains unchanged

**Fig. 5:**
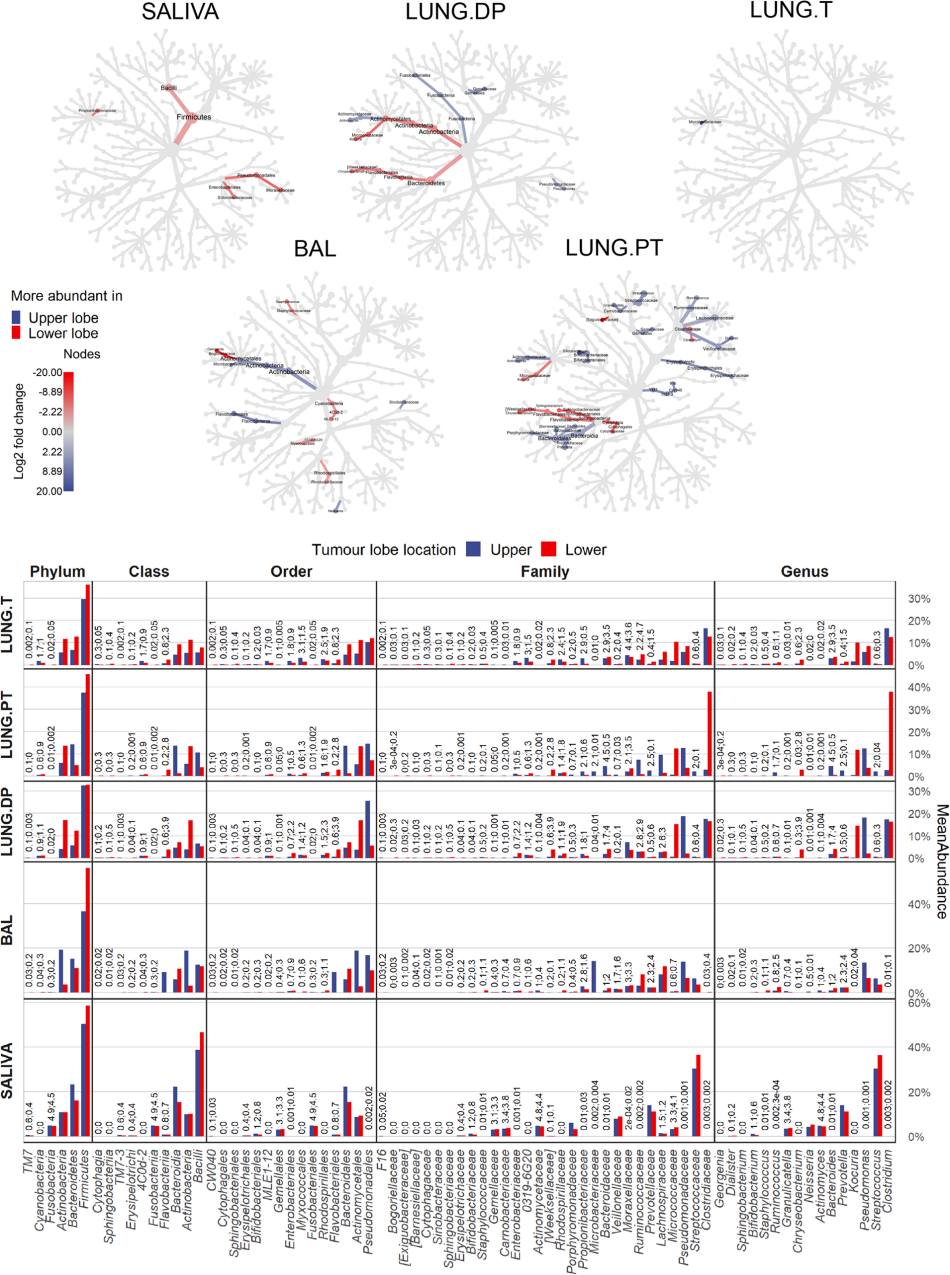
Differential abundance between upper and lower tumour lobes in salivary and lung microbiota. Each tree represents taxa with significantly different abundance relative to sample’s origin (for lung) in the upper or lower tumour lobe. For saliva, the comparison shows the significant difference in salivary microbiota between patients with tumour either in upper or lower lobe. Coloured nodes and branches represent the taxa with significantly different abundance and the intensity is proportional to log2-fold change in abundance in the favour of the lobe noted with the same colour. Statistical significance was assessed by zero-inflated general linear model using Wald’s test (DESeq), with p-value threshold of α ≤ 0.05 after BH correction. Bar chart shows the relative abundance of taxa noted in the taxonomical trees. BAL - bronchoalveolar lavage fluid, LUNG.DP - non-malignant distal piece, LUNG.PT - peritumoural tissue, LUNG. T - tumour

Tumour location in UL or LL significantly influenced the microbial abundance in each of the analysed sample types, but not in the same manner (Fig. 5). As suggested by diversity results, microbiota of the peritumoural tissue seemed to be the most influenced by the lobe location. The changes were limited to members of the three major phyla: *Firmicutes*, *Actinobacteria* and *Bacteroidetes*, and candidate phylum *TM7*. More precisely, in UL peritumoural tissue more abundant were members of the phylum *Firmicutes*, phylum *TM7,* classes *Bacteroidia* (phylum *Bacteroidetes*) and two families from phylum *Actinobacteria* (*Actinomycetaceae* and *Bifidobacteriaceae*). This abundance pattern was very similar to pattern of initial comparison between BAL and each of the lung tissue samples (Fig. 2a). This, added to the results of beta diversity, could confirm that microbiota in UL is more similar between peritumoural tissue and BAL then it is in the lower lobes. In the LL, peritumoural tissue microbiota was enriched with three classes from phylum *Bacteroidetes* (*Flavobacteriia*, *Sphingobacteriia* and *Cytophagia*), and with families *Clostridiaceae* (genus *Clostridium*) and *Micrococcaceae* (genus *Kocuria*) from phylum *Firmicutes* and *Actinobacteria*, respectively.

Compared to number of affected taxa in peritumoural tissue, both BAL and non-malignant tissue were less influenced by the lobe location, while tumour seemed to be almost entirely unaffected (Fig. 5). Moreover, phylum *Firmicutes* and class *Bacteroidia*, which in peritumoural tissue had the highest number of members with significant differences, were not found significantly different in either non-malignant tissue or BAL. Instead, UL non-malignant tissue was enriched with the phylum *Fusobacteria* and only a few other lower taxa without involvement of the whole clades (order *Gemellales*, families *Actinomycetaceae* and *Pseudomonadaceae*)*.*

Interestingly, in both non-malignant and peritumoural tissue, phylum *Actinobacteria* (with genus *Kocuria*) and class *Flavobacteriia* (with genus *Chryseobacterium*) were significantly more abundant in LL compared to UL. However, opposite from the two tissues, in BAL these same two taxonomic groups were significantly more abundant in UL (Fig. 5). Instead of having a common abundance value in one lobe and a lower or higher value in the other lobe, the abundances between BAL and tissue samples were inverse in each lobe. E.g. the abundance of clade *Actinobacteria* in BAL and tissues was 19% versus ~ 5% in upper lobes, and 4% versus ~ 13% in lower lobes, respectively). Except the mentioned two clades of *Actinobacteria* and *Flavobacteriia*, no other larger taxonomic groups were found as more abundant in UL BAL. Similarly, taxa found as more abundant in the lower lobes were dispersed between different phyla (*Cyanobacteria*, members of *Proteobacteria*, *Firmicutes*, and *Actinobacteria*).

Even though importance was given to log2 fold change of abundance, certain taxa held however high impact on the overall composition of the samples due to their higher relative abundance (Fig. 5, Additional file: Figure 1). This was particularly true for three genera, *Clostridium, Kocuria* and *Pseudomonas*. In peritumoural tissue, genus *Clostridium* (phylum *Firmicutes*) ranged from 3% in UL vs 38% in LL, in non-malignant tissue genus *Pseudomonas* (*Gammaproteobacteria*) represented 18% in UL vs 2% in LL lobes, and genus *Kocuria* (phylum *Actinobacteria*) ranged from 0.5 and 0.2% in UL to 12 and 15% in LL in peritumoural and non-malignant tissue, respectively.

Finally, saliva samples, as the extrapulmonary sample with no direct physical connection to the tumour location as lung microbiota, also showed significantly different abundance profile relative to tumour lobe location. If tumours were found in LL, saliva was significantly enriched in class *Bacilli* and families *Enterobacteriaceae*, *Moraxellaceae* (both *Gammaproteobacteria*) and *Propionibacteriaceae* (*Actinobacteria*). Curiously, no taxa were detected as significantly more abundant if tumour was found in UL.

### Stratification between lobes defines differences between BAL and tissues and confirms similarity of BAL and peritumoural tissue in upper lobes

**Fig. 6:**
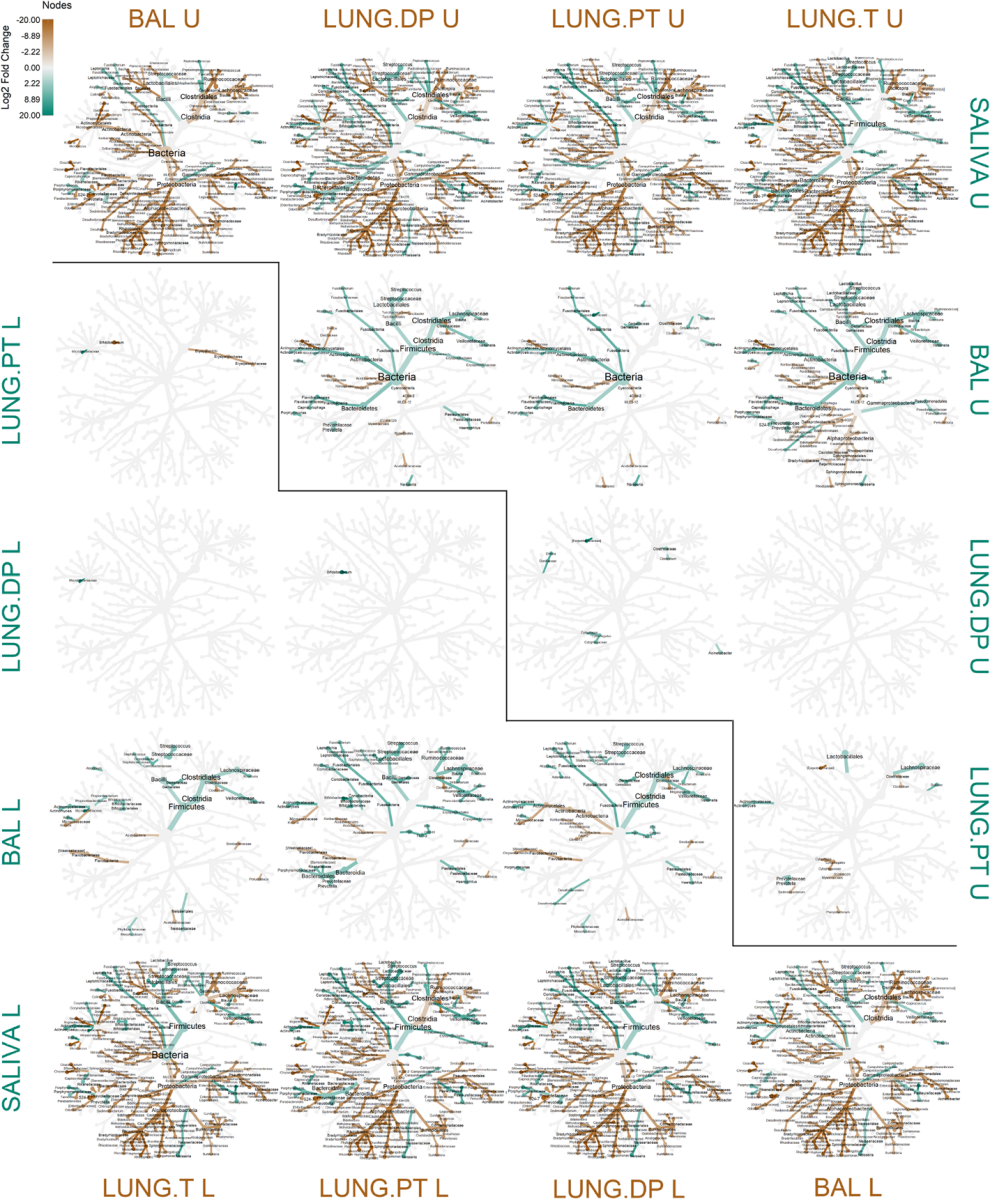
Comparison of abundance between salivary and lung microbiota relative to tumour lobe location. The two parts of the figure represent comparison in abundance between samples linked to upper (U) lobes in the upper part of the figure and to lower (L) lobes in the lower part of the figure. Coloured nodes and branches represent taxa with significantly different abundance between the two compared samples. The colour intensity is proportional to log2-fold change in abundance in the favour of the sample with the same colour. Statistical significance was assessed by zero-inflated general linear model using Wald’s test (DESeq), with p-value threshold of α ≤ 0.05 after BH correction. BAL - bronchoalveolar lavage fluid, L – lower lobe, LUNG.DP - non-malignant distal piece, LUNG.PT - peritumoural tissue, LUNG. T – tumour, U – upper lobe

Stratification of samples by lobe location also revealed or defined differences between samples (Fig. 6). Existence or disappearance of certain differences was a direct result of individual lobe-related characteristics reported in the Fig. 5. Between saliva and the four lung samples, there was little change (Fig. 6), since these samples were initially already very different (Fig. 3). Several taxa were no more significant between saliva and BAL from upper lobes, while they were significant in all other comparisons (e.g. *Nitrospirae, Neisseriales, Haemophilus*). Comparing saliva and the three lung tissues, only taxa *Spirochaetes, Sphingobacteria* and *TM7* showed changes relative to the lobe location. Among them, class *Sphingobacteria* was significantly more abundant in all lung tissues when compared to saliva, except in UL peritumoural tissue. This was interesting because neither UL nor LL BAL showed this difference, adding to similarity of BAL and peritumoural tissue in UL. Despite these changes and stratification by location, saliva was still predominantly abundant in *Firmicutes, Fusobacteria, Epsilonproteobacteria, Erysipelotrichi, Prevotella* and *Neisseria* clade, while lung samples dominated in abundance of *Proteobacteria, Acidobacteria, Nitrospirae, Verrucomicrobia, Cyanobacteria*, and the rest of *Bacteroidetes* (except *Prevotella* clade).

Unlike the comparison between saliva and lung samples, the aspect of lobe location induced much more developed and varying differences between BAL and tissues. Similarly to comparison with saliva, differential abundance pattern varied for both *Nitrospirae* and *Cyanobacteria* between BAL and each of the three tissues relative to lobe location. Further, as expected, *Flavobacteriia* and *Actinobacteria* showed inverse abundance between the two, being both more abundant in UL BAL and in LL tissues (Figs. 5 and 6). Interestingly, differences in phyla *TM7* and *Acidobacteria* came in pairs. The significantly increased abundance of *TM7* in BAL was accompanied with increased abundances of *Acidobacteria* in the tissues, otherwise no difference was seen in either of them. In UL, this was seen in tumour, while in LL this was seen for peritumoural and non-malignant tissue.

Interestingly, the pattern of differential abundance was much more similar between different comparisons of BAL and tissues in the lower lobes than in the upper lobes. Here, the highest number of differences was found between tumour and BAL, and unlike in other tissues, included a large portion of phylum *Proteobacteria*, especially class *Alphaproteobacteria,* as more abundant in tumour. Also, peritumoural tissue involvement of members from the phylum *Firmicutes* was minimal compared to other two tissues, excluding an important differentiating factor. This could be, therefore, one of the major reasons behind increased similarity between BAL and peritumoural tissue in UL. Detailed individual differences between BAL and tissues can be found in Additional file 1: Text 1 “Additional explanation of differences between lung samples”.

Finally, significant differences were noted between tissues (Fig. 6), but they were, however, few in number. They were dispersed within four most abundant phyla and considered mostly endpoint taxa. Also, there were less differences in LL than in UL. Curiously, in both locations, more difference was found between tumour and peritumoural tissue, than between tumour and non-malignant tissue (none and one in UL and LL, respectively). Looking at the three tissues, in upper lobes class *Cytophagia*, family *Exiguobacteraceae* and *Clostridiaceae* were the least abundant in peritumoural tissue, while in LL family *Microbacteriaceae* had significantly the lowest abundance in tumour. Detailed individual differences between the tissues can be found in Additional file 1: Text 1 “Additional explanation of differences between lung samples”.

## Discussion

Following the last advancement on the interaction between gut microbiota, immune system and the tumour environment [8–11], there has been a growing interest in studying this concept in other physiological environments involving extraintestinal tumours. However, before exploring the effect of the gut microbiota, there has been an increasing necessity to investigate the effect of the local microbiota on the tumour as well [42]. Despite the emerging number of studies on the lung microbiota in different malignancies [17, 43–46], its involvement in lung cancer is in its promising beginnings [26, 30, 32, 33, 47]. However, for the moment there is still no study considering the ground difference between different lung samples and their microbiota, while it is suggested that those could harbour microbiota with varying characteristics [21], and therefore, have diverging interactions with local immunity and tumour.

This study is, to our knowledge, the first to characterise the lung microbiota originating from four different lung samples (BAL, non-malignant tissue, peritumoural tissue and tumour), accompanied with the characterisation of salivary microbiota in NSCLC patients. We hypothesised that samples’ nature, “architecture”, physiological functions and environment, will influence characteristics of associated lung microbiota. Therefore, BAL should represent “planktonic” bacterial population found within the bronchial lumen or associated with biofilms or mucus [48], sampled along with the hydrodynamic force of the instilled liquid. Non-malignant tissue from the same lobe but taken on the opposite side from the tumour should represent a sample with a normal lung architecture, with well-defined small alveolar spaces and single-cell epithelial layer. In majority, it should harbour the biofilm-, mucus- and cell-associated lung microbiota. On the contrary, tumour represents a tissue with disrupted architecture, varying in form and obstruction degree relative to its type and grade [2]. Tissue modelling could also involve overproduction of the mucus as seen in certain subtypes of adenocarcinoma [49], but also different reaction of the immune system [50]. Finally, peritumoural tissue represents a non-malignant tissue in the direct contact with the tumour, separated as based on different histological properties. In literature it is addressed as tumour microenvironment and harbours different roles in stimulation or suppression of tumour metabolism [51]. Therefore, we presumed that its characteristics would be different both from the distal non-malignant tissue and the tumour. Except for the number of different lung samples, the particularity of this study is also the way of obtaining BAL. It was obtained directly on the excised lobe containing tumour without using the bronchoscope, to minimise contamination risk of upper airways and increase the precision in characterisation of “true” BAL microbiota in the tumour proximity.

We reported that the four lung samples significantly clustered versus oral microbiota based on beta diversity, a confirmation of a previous result in healthy subjects that lung microbiota is distinct from other communities [32]. Salivary microbiota had several lower alpha diversity metrics compared to lung microbiota, as well as high dominance of genus *Streptococcus* and overall phylum *Firmicutes*. Along with its homogenous core microbiota (again prevalence of *Streptococcus* OTUs) that represented almost ¾ of total relative abundance per patient, this indicated its inter-subject stability and lower complexity compared to the one of the lung samples. In addition, the significant difference in abundance including the majority of the taxa detected in samples clearly separated the four lung samples versus oral microbiota. We identified typically oral taxonomic groups more abundant in saliva in all comparisons, such as *Fusobacteria, Spirochaetes, Synergistetes, Erysipelotrichi, Epsilonproteobacteria, Bacilli* and *Neisseriales*, most of them being in concordance with previous literature [52, 53]. On the other hand, phyla *Acidobacteria*, *Cyanobacteria, Nitrospira, Verrucomicrobia* and *Planctomycetes* were detected as strictly lung-associated (detected in each lung sample) with no representatives in saliva.

Further, we showed that BAL microbiota, even though undoubtedly belonging to lung microbiota cluster, had significantly different features from tissues. First, multidimensional representation of the beta diversity placed BAL samples on the side of the lung cluster towards salivary microbiota (Fig. 1a). Even though presence of more abundant salivary taxa was very limited in both tissue samples and BAL, *Streptococcus, Prevotella* and *Veillonella* (the three most abundant genera in saliva) were found elevated in BAL compared to tissue samples. It was previously suggested that these and other typically oral bacteria are found in low abundance in healthy lungs, due to their constant elimination [54]. Since BAL represents microbiota of the bronchial lumen, which undergoes constant influx of upper airways particles by respiration and is also the first “space” influenced by microaspiration [55], those could explain increased presence of these supraglottic taxa in BAL compared to the lung tissues. This position “in the middle” was also seen when looking at the differential abundance between all five samples (Fig. 5). The taxa that were more abundant in saliva when compared to the four lung samples almost perfectly corresponded to taxa that were more abundant in BAL when compared to the three lung tissues. Second, lung samples had *Proteobacteria* and *Firmicutes* as the two most abundant phyla, however with different ratios. While BAL was dominated by *Firmicutes*, almost twice as abundant as *Proteobacteria*, lung tissues were dominated by *Proteobacteria* (as previously reported [32]), or at best, had equal abundance of the two (peritumoural tissue). Here it is however important to note that the high abundance of phylum *Firmicutes* in lung samples was due to members of the class *Clostridia*, unlike in saliva, where it was due to highly abundant class *Bacilli*. This emphasises one of the essential differences between oral and lung microbiota often omitted by selective presentation of only phylum or genus level. A third feature was that BAL did not differentiate from lung tissues only by difference in taxa abundance, but also by the presence of BAL-specific bacteria, such as genus *Capnocytophaga,* or absence of tissue specific-bacteria, such as genus *Kocuria* or *Clostridium*. The two latter genera were, however, detected in BAL, but with relative abundance 175-fold and 520-fold lower (~ 0.03%), respectively, than in the tissues. This detection is possibly due to their presence in host’s cells during the DNA extraction, since cellular BAL was used for BAL analysis [48]. Lastly, no differences in alpha diversity metrics were detected between four lung samples, suggesting two different but equally “rich” lung microbiota populations. All this supports the hypothesis that BAL indeed represents a unique lung microbiota in lung cancer and that concerns of diverging results due to different samples have been justified [21].

Conversely to our hypothesis, there was no significant difference detected between the three lung tissues when analysing the totality of samples. Although increased diversity of non-malignant tissue versus tumour has been previously suggested [32], we did not find the same. This could be due to the fact that our study group was more balanced in patients with less and more advanced tumour stages. In a referenced study, the majority of subjects had tumours in stages I and II, showing decreased diversity compared to higher stages according to authors.

We also reported on the existence of the core OTUs and that these were mostly shared between lung tissues and partially with BAL, consisting in the majority of the members of *Proteobacteria*. Interestingly, one of the core OTUs was uniquely associated to species *Variovorax paradoxus*, an ambiguous aerobic organism found in many niches, also in oral microbiome, and known for its potent biofilm creation [56, 57]. It was detected as the core OTU in all four lung samples, while conversely, it was not found in saliva (where it was originally detected). This could suggest its potential role in biofilm creation in lower airways and would be an interesting topic for future verification and investigation of potential role in pathogenesis of lung cancer [58].

Even though still under debate, several studies connected lower lobe tumours with worse prognostics [5, 59, 60]. Therefore, we examined the characteristics of the lung, but also salivary microbiota, if the tumour was found in upper or lower lobes. Indeed, we noted several changes in alpha and beta diversity but they were focused on changes in BAL and peritumoural tissue (with lower and higher alpha diversity, respectively, in LL compared to UL). We reported that in all four lung samples, lower lobes had decreased abundance of phylum *Proteobacteria* into favour of *Firmicutes*. Increased abundance of phylum *Firmicutes* was previously seen in BAL of LC patients compared to patients with benign-mass lesions [47], but also in patients with COPD [21]. In our study, 62% of patients in the LL were diagnosed with stage III tumours, against 30% in the UL. Even though this remains a small study, this finding supports that “shift” from *Proteobacteria* to *Firmicutes* might have a role in lung cancer progression.

We showed that location significantly influenced the abundance of two clades, *Actinobacteria* and *Flavobacteriia*, in the inverse manner between BAL and extratumoural tissues. While both are more abundant in UL BAL, their abundance in UL extratumoural tissues was 2–10-fold lower, with exact inverse situation in LL. Both genus *Chryseobacterium* sp. (*Flavobacteriia*) and *Kocuria rhizophila* (*Actinobacteria*) detected as more abundant in LL tissues have been previously reported as uncommon human pathogens [61, 62]. However, their increased presence selectively in LL tissues and their important overall abundance (~ 4 and 12%, respectively) suggest that there might be an important communication between different lung environments depending on local conditions or malignancy status, influencing preferential bacterial growth in one type of considered environment.

Interestingly, we found significant differences in abundance of salivary microbiota between patients with tumour in either upper or lower lobe. All differences considered taxa elevated in the case of LL tumours and included families previously associated with bacterial exacerbations and infections (*Moraxellaceae*, members of *Bacilli* as *Streptococcus, Staphylococcus*, etc.) [63] or pulmonary complications (*Propionibacteriaceae, Enterococcaceae*) [64–66]. However, these taxa were not found elevated in LL lung microbiota. This, however, does not exclude their implication in malignant changes or being its consequence, and will be an interesting topic for further consideration.

Finally, we found that peritumoural tissue showed higher similarity to BAL in UL both in beta diversity and in abundance, while in LL it shared characteristics with other tissue samples. Moreover, we found that alpha diversity of peritumoural tissue increased in LL to the level of other tissues, while the ones of BAL decreased. Peritumoural tissue is the tissue in direct contact and interaction with the tumour. In the history of cancer research, the role of extracellular matrix (ECM) surrounding tumour has been extensively studied [67]. Tumour cells are found to be able to directly influence the rearranging of connective units (such as collagen) and degradation of ECM to create a tumour-permissive environment and enabling metastatic progression [68–70]. In our case, it is possible that the observed changes in peritumoural tissue composition reflect certain remodelling of ECM, which could therefore be either more or less permissive for microbial attachment.

Curiously, of all samples, tumour was almost completely uninfluenced by location. Rather than from the outside, it is possible that intratumoural conditions, such as oxygen availability, density, necrosis and other factors [71, 72] independent of the external conditions are more likely to be the influencing ones. This is, however, a matter for further research.

The major strongpoint of this study is the analysis of the microbiota from four different lung samples covering the major physiologically different environments in NSCLC patients that has not been characterised previously, with the addition of saliva as the sample of oral microbiota. The representation using taxonomical trees also gives better insight into sample’s composition, especially important in characterisation of this type of as yet, scarcely defined microbiota. Moreover, this study reports higher numbers of detected OTUs as well as diversity indexes than previously suggested for saliva, non-malignant tissue, tumour and BAL [29, 32, 33]. The reason could be due to the higher quality of obtained samples (only 6 from 85 samples lower than 1000 reads) and direct sampling of the BAL in the excised lobe. Also, the observed study group and stratification by tumour lobe location were well balanced in various clinical and demographic factors, minimising the result’s bias.

The major drawback of this study is a low number of subjects, due to strict inclusion criteria for the overall protocol and a limited recruiting time. Additional inconvenience is that, even though initially requested, forming a control group has not been authorised by the Ethics Committee due to the invasiveness of the sampling techniques. Certain studies on lung cancer microbiota have adapted different strategy to include a non-malignant control group in their protocols, e.g. prospective studies analysing BAL from patients with pulmonary nodules that are next characterised as either benign or malignant (i.e. lung cancer) [47]. However, patients diagnosed with benign pulmonary nodules represent the minority, in most cases they do not require any treatment and partial resection is undertaken only in rare occasions [73, 74]. With the objective to analyse the lung microbiota from multiple lung samples (BAL in situ and different tissues), the partial resection in benign lung nodules would exclude both the possibility of performing BAL directly on the excised lobe and obtaining different types of lung tissue relative to the distance from the nodule. Moreover, it is unclear for the moment whether the presence of benign pulmonary nodule also modifies the local lung microbiota, introducing a potential bias in interpretation without previous investigation. Considering other surgically treatable lung diseases, such as interstitial lung disease [75], collapsed lung [76], emphysema or bronchiectasis [77], they represent malignancies with a distinct physiological condition and were not considered for use as a potential control group. The ideal control group would be the one including lobectomy in “healthy” patients, as seen in certain transplantation cases [78].

The limiting factor was also the decision to obtain BAL directly from the excised lobe to improve the precision in characterisation, but disabling the possibility of sampling the complementary non-tumour lobe by bronchoscopy due to difference in sampling technique.

Finally, it is important to note that the surgical resection considers fewer than 20% of all lung cancer patients and includes less severe cases [79]. Therefore, our results cannot be extrapolated to the overall lung cancer community, especially the most advanced cases relying mostly on multimodality treatment [2].

## Conclusion

To our knowledge, this is the first trial that studied oral and lung microbiota from both BAL and three different tissue samples in 18 NSCLC patients. We confirmed that oral and lung microbiota are significantly different both in diversity and in taxonomy. However, we showed that BAL indeed represents a unique microbiota compared to three different tissue microbiota (from non-malignant, peritumoural and tumour tissue). We found that location of the tumour in upper or lower lobes influenced both oral, BAL and extratumoural microbiota with detection of *Firmicutes* as dominating phylum, but surprisingly, not the one of the tumours. Moreover, few differences were found between tissues, suggesting that these are not conditioned by lobe location and turning attention to other factors for future consideration, such as tumour type, aggressiveness or metastatic changes. Finally, the most intensive changes in microbiota relative to location were seen in peritumoural tissue, possibly reflecting changes in tumoural ECM. Our findings are the first to give essential characteristics and differences within lung microbiota of NSCLC and their susceptibility to tumour lobe location, proposing several possible implications of microbiota in pathology of lung cancer and suggesting potential research directions for better understanding of the lung microbiota-cancer interaction.

## Supplementary information

**Additional file 1: Figure 1.** Most abundant genera in four lung and salivary microbiota relative to tumour lobe location. **Text 1.** Chapter “Additional explanation of differences between lung samples (main manuscript: Fig. 6)” that adds details on significant differences found in Fig. 6.

## Data Availability

According to the practice of the sponsoring institution (Centre Jean Perrin), all samples will be preserved for 15 years. The datasets generated and/or analysed during the current study are confidential but are available from the corresponding author on reasonable request and after the data sharing process is brought into compliance with GDPR (Regulation (EU) 2016/679 of the European Parliament and of the Council of 27 April 2016).

## Abbreviations

ADC: Adenocarcinoma
BAL: Bronchoalveolar lavage fluid
BH: Benjamini-Hochberg
BMI: Body mass index
COPD: Chronic obstructive pulmonary disease
DLCO: Predicted diffusing capacity of the lung for carbon monoxide
ECM: Extracellular matrix
FEV_1_: Forced expiratory volume per second
FVC: Forced vital capacity
KW: Kruskal-Wallis test
LC: Lung cancer
LL: Lower lobe
LUNG.DP: Non-malignant distal lung piece
LUNG.PT: Peritumoural lung tissue
LUNG.T: Tumour tissue
NMDS: Non-metric multidimensional scaling
NSCLC: Non-small cell lung cancer
OTU: Outer taxonomic unit
PD-1: Programmed cell Death protein 1
QIIME: Quantitative Insights Into Microbial Ecology
SCC: Squamous cell carcinoma
T: Tumour
UL: Upper lobe
uwUF: Unweighted UniFranc distance
wUF: Weighted UniFrac distance
