# Supplementary material for: Characterisation of microbiota in saliva, bronchoalveolar lavage fluid, non-malignant, peritumoural and tumour tissue in non-small cell lung cancer patients: a cross-sectional clinical trial
Source: Respir Res. 2020 May 25;21:129. doi: 10.1186/s12931-020-01392-2 (PMC7249392; doi:10.1186/s12931-020-01392-2)
Supplement: Supplementary file 1 — Additional file 1: Figure 1. Most abundant genera in four lung and salivary microbiota relative to tumour lobe location. Text 1. Chapter “Additional explanation of differences between lung samples (main manuscript: Fig. 6)” that adds details on significant differences found in Fig. 6. [file 12931_2020_1392_MOESM1_ESM.pdf]

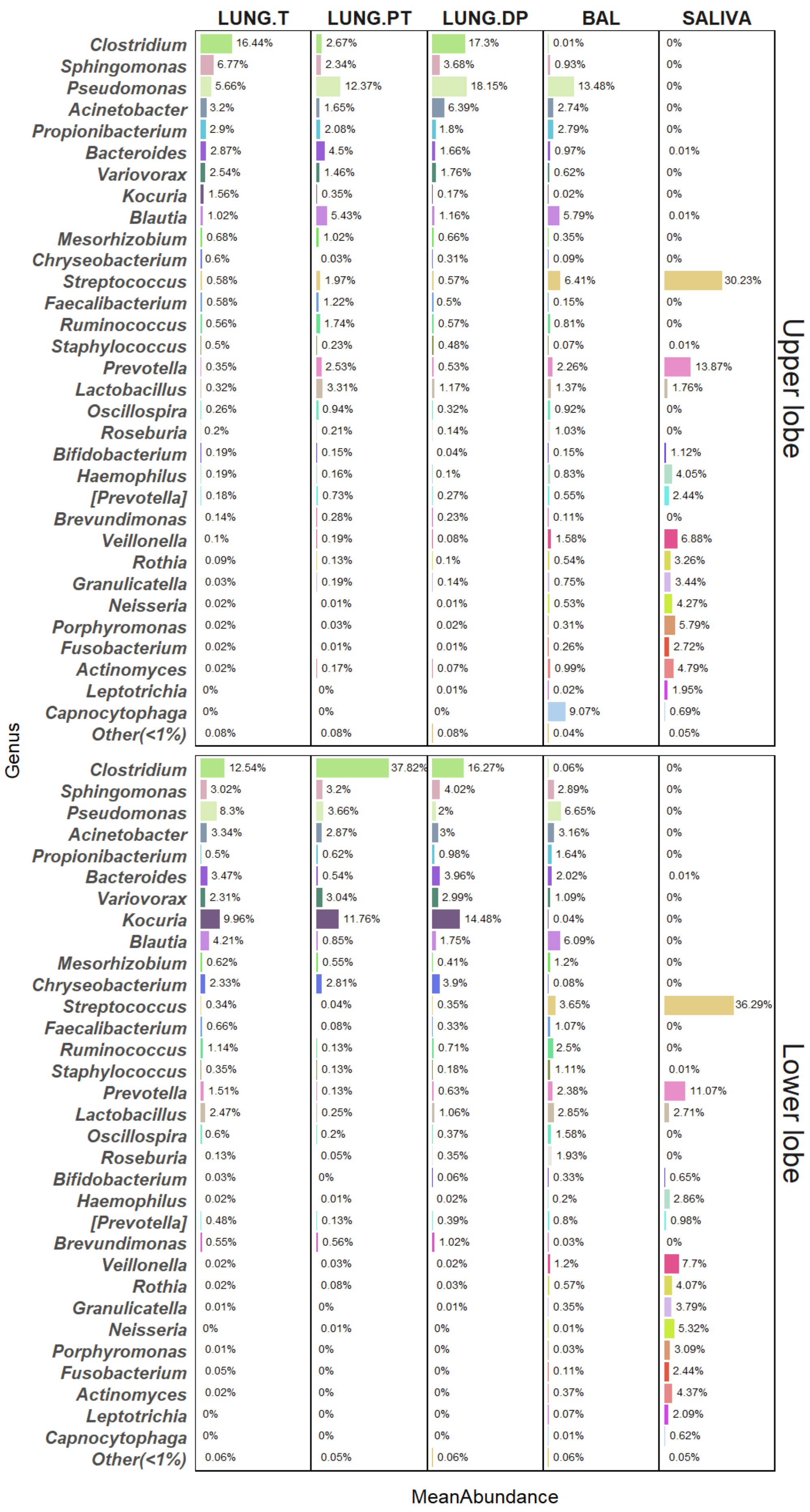

Figure 1 Most abundant genera in the four lung and salivary microbiota relative to tumour lobe location. BAL - bronchoalveolar lavage fluid, LUNG.DP - non-malignant distal piece, LUNG.PT - peritumoural tissue, LUNG.T - tumour.

Text 1. Additional explanation of differences between lung samples (main manuscript: Fig. 6)

Except mentioned characteristics, non-malignant tissue and BAL shared similar differences between locations, unlike other two tissues. Peritumoural tissue and BAL were more different in lower (LL) than in upper lobes (UL), seen by significantly increased abundance of members of class *Bacteroidia*, *Coriobacteriia*, overall phylum Firmicutes and orders *Pasteurellales, Bifidobacteriales* in LL BAL than in LL peritumoural tissue, not seen in UL. On the other hand, tumour and BAL were more different in UL and represented the richest comparison tree in tissue vs. BAL category (Main manuscript: Fig. 6). Classes *Alphaproteobacteria, Cytophagia, [Saprospirae]* and orders *Myxococcales, Bdelovibrionales* and Turicibacterales were significantly more abundant in UL tumour than in BAL, and no difference was seen in LL. On the contrary, phylum *Fusobacteria* and class Gammaproteobacteria were both more abundant in UL BAL than in tumour (no diff. in LL).

Additionally, genera *Acinetobacter* and *Dietzia* were more abundant in non-malignant tissue than in peritumoural tissue, while no differences were detected between non-malignant tissue and tumour in UL. Conversely, several taxa were different between UL peritumoural tissue and tumour, including family *Lachnospiraceae* and genera *Actinomyces, Dialister* and *Prevotella* more abundant and *Sediminibacterium, Phenylobacterium* and family *Myxococcales* less abundant in UL peritumoural tissue. In LL, the differences were very few. Genus *Bifidobacterium* was significantly less abundant in LL peritumoural tissue in the three tissues and family *Microbacteriaceae* in tumour. In addition, class *Erysipelotrichi* was significantly more abundant in LL tumour than in LL peritumoural tissue.
